# New Hybrid Scaffolds Based on 5-FU/Curcumin: Synthesis, Cytotoxic, Antiproliferative and Pro-Apoptotic Effect

**DOI:** 10.3390/pharmaceutics15041221

**Published:** 2023-04-12

**Authors:** Gustavo Moreno-Quintero, Emmanuel Betancur-Zapata, Angie Herrera-Ramírez, Wilson Cardona-Galeano

**Affiliations:** Chemistry of Colombian Plants Group, Institute of Chemistry, Faculty of Exact and Natural Sciences, University of Antioquia, Calle 70 No. 52–21, A.A 1226, Medellín 050010, Colombia

**Keywords:** 5-FU, curcumin, hybrid, cytotoxicity, antiproliferative, colorectal cancer, apoptotic effect

## Abstract

A series of 5-FU-Curcumin hybrids were synthesized, and their structures were elucidated by spectroscopic analysis. The synthesized hybrid compounds were evaluated in different colorectal cancer cell lines (SW480 and SW620) and in non-malignant cells (HaCaT and CHO-K1), to determine their chemopreventive potential. Hybrids **6a** and **6d** presented the best IC_50_ value against the SW480 cell line with results of 17.37 ± 1.16 µM and 2.43 ± 0.33 µM, respectively. Similarly, compounds **6d** and **6e** presented IC_50_ results of 7.51 ± 1.47 µM and 14.52 ± 1.31 µM, respectively, against the SW620 cell line. These compounds were more cytotoxic and selective than curcumin alone, the reference drug 5-fluorouracil (5-FU), and the equimolar mixture of curcumin and 5-FU. In addition, hybrids **6a** and **6d** (in SW480) and compounds **6d** and **6e** (in SW620) induced cell cycle arrest in S-phase, and, compounds **6d** and **6e** caused a significant increase in the sub-G0/G1 phase population in both cell lines. Hybrid **6e** was also observed to induce apoptosis of SW620 cells with a respective increase in executioner caspases 3 and 7. Taken together, these results suggest that the hybrids could actively act on a colorectal cancer model, making them a privileged scaffold that could be used in future research.

## 1. Introduction

Colorectal cancer (CRC) is the second-deadliest and most widely diagnosed cancer in the world, accounting for 10% of all cancers. This pathology presents a great geographical distribution, and the patterns are very similar among men and women, being the third most common cancer in men and the second in women. Among the risk factors that contribute to CRC development, it is possible to include alcohol consumption, smoking, physical inactivity, obesity, diets high in fat and red meat, and inadequate intake of dietary fiber, vegetables, and fruits. Due to the widespread occurrence of the risk factors and the increase in the statistics, extensive research is ongoing to develop new pharmaceutical agents with chemopreventive potential against CRC [1].

5-fluorouracil (5-FU) is one of the major anticancer agents used clinically for the treatment of several kinds of cancer, including CRC. However, this compound not only exhibits a short half-life and low selectivity but also various toxic side effects [2]. For these reasons, it has been modified in different ways to obtain conjugates and hybrid molecules, which may improve its therapeutic index and reduce the side effects [3]. Curcumin is one of the bioactive components of Curcuma longa Linn. Several studies with this compound have revealed important functions in cancer control [4], including antiproliferative activity against different cancer cell lines, inhibition at different stages of cancer cell progression [5], and its effect on a variety of growth factor receptors and cell adhesion molecules involved in tumor growth, metastasis, apoptosis, and multidrug resistance [6,7,8].

Molecular hybridization is a promising approach for the synthesis of new anticancer compounds, consisting of the chemical combination of two or more molecules with biological activity [9,10,11]. One of the main advantages of this strategy is that it not only synergizes their biological effect but also increases their ability to inhibit more than one biological target. The goal of the hybrid molecule approach is to improve anticancer activity and selectivity while causing an important reduction in side effects [12].

**Figure 1:**
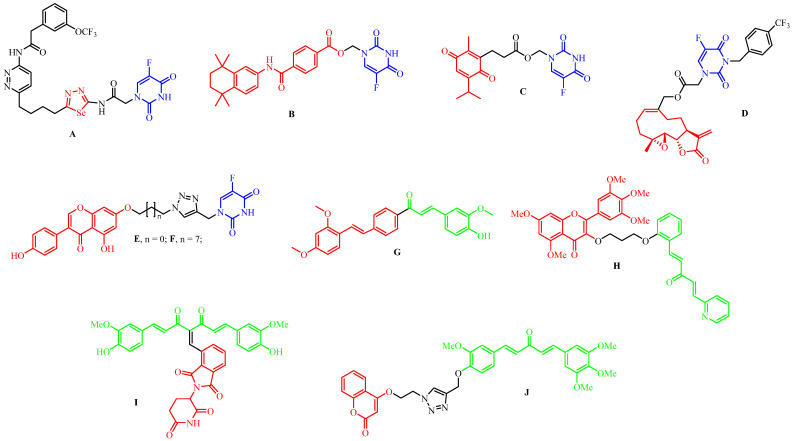
Hybrid molecules derived from 5-FU and curcumin with anticancer activity.

5-FU-1,3,4-Selenadiazole hybrid A displayed good activity over the A549 cell line with IC_50_ values of 0.02 and 0.46 µM [13]. 5-FU-Tamibarotene hybrid B exhibited activity over U937, HL-60, and K562 cell lines with an IC_50_ of 8.21, 9.87, and 10.64 µM, respectively. This compound displayed better activity than 5-FU and tamibarotene [14]. Hybrid C (5-FU- Thymoquinone) was effective in HCT116 cells with an IC_50_ value of 16 µM. The cytotoxic effects of C against the HCEC non-malignant corneal epithelial cells were lower in comparison to tumor cell lines, as observed by the increase in the IC_50_ value (IC_50_ = 52 µM), which suggests this compound is selective to cancer cells [15]. 5-FU-Parthenolide hybrid (D) exhibited the highest activity against both cell lines, but it was lower than Adriamycin (IC_50_ = 0.73 and 1.14 µM against Bel7402 and Bel-7402/5-FU, respectively). Compound D exhibited cytotoxicity against normal 3T3 cells (mouse embryonic fibroblast cell line) with an IC_50_ value of 5.2 µM, showing a therapeutic index value of 2.3. Preliminary molecular mechanisms showed that this hybrid could induce apoptosis of Bel-7402/5-FU cells through the mitochondria-mediated pathway and reverse drug resistance by inhibiting MDR1, ABCC1, and ABCG2 to increase intracellular drug accumulation [16]. Hybrid E was active on SW480 and SW620 cells (IC_50_ = 62.73 ± 7.26 µM and 50.58 ± 1.33 µM, respectively), while compound F showed activity only on SW620 (IC_50_ = 36.84 ± 0.71 µM). These hybrids displayed better selectivity than genistein and 5-FU. In addition, hybrids E and F exhibited anti-proliferative activity and cell cycle arrest at the S-phase and G2/M, and compound E induced apoptosis in SW620 cells [17] (Figure 1).

The chemopreventive potential of hybrids based on curcumin and resveratrol was evaluated against SW480 human colon adenocarcinoma cells, its metastatic derivative, and the SW620 cell line. Hybrid G displayed the best cytotoxic activity in both cell lines, with IC_50_ values of 29.18 µM for SW480 and 20.44 µM for SW620 [18]. Curcumin-chromone hybrid H was evaluated for its cytotoxic activity against human liver cancer cells (SMMC-7721), human gastric cancer cells (SGC-7901, MGC-803), and human glioma cells (U87). This compound displayed IC_50_ values of 1.08, 0.56, 3.18, and 3.93 µM over SGC-7901, MGC-803, SMMC-7721, and U87, respectively. Moreover, this hybrid showed that it could induce apoptosis in gastric cancer cells by arresting the cell cycle, breaking mitochondrial function, and inhibiting TrxR activity [19]. A hybrid molecule based on curcumin and thalidomide I was tested on human multiple myeloma MM1S, RPMI8226, and U266 cells. The biological results showed that this compound exhibited significantly improved lethal effects towards all three human MM cell models (IC_50_ = 4.55, 6.44, and 3.96 µM against RPMI8226, MM1S, and U266 cells, respectively). Furthermore, mechanistic studies in U266 cells demonstrated that compound I can induce the production of reactive oxygen species (ROS) and cause G1/S arrest, thus leading to apoptosis and cell death [20]. Hybrid J, which is based on curcumin and coumarin linked by a triazole ring, showed significant cytotoxic potential against THP-1, COLO-205, and HCT-116 cell lines with IC_50_ values of 0.82, 4.68, and 2.21 µM, respectively. This compound was found to significantly inhibit tubulin polymerization (IC_50_ = 0.82 μM in THP-1 tumor cells) [21] (Figure 1).

**Figure 2:**
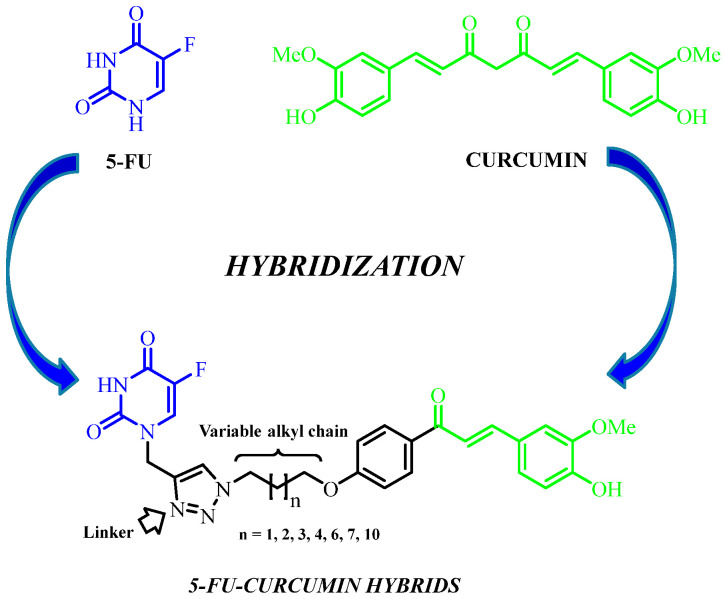
Design of 5-FU-Curcumin hybrids.

Considering the background described above and the urgent need to develop new therapeutic alternatives for the treatment of colorectal cancer, we synthesized several hybrids based on 5-FU and curcumin via the click reaction as a key step in the synthesis (Figure 2). Besides, we evaluated their biological activity using colon adenocarcinoma cells (SW480) and their metastatic derivative (SW620) to determine the chemopreventive potential against this type of cancer.

## 2. Materials and Methods

### 2.1. Chemical Synthesis

#### 2.1.1. General Remarks

Microwave reactions were carried out in a CEM Discover microwave reactor in sealed vessels (monowave, maximum power 300 W, temperature control by an IR sensor, and fixed temperature). ^1^H and ^13^C NMR spectra were recorded on a Varian instrument operating at 300 and 75 MHz, respectively. The signals of the deuterated solvent (CDCl_3_ or DMSO-D6) were used as references. Chemical shifts (δ) are expressed in ppm with the solvent peak as a reference and TMS as an internal standard; coupling constants (J) are given in Hertz (Hz). HRMS was obtained using a Bruker Impact II UHR-Q-TOF mass spectrometer (Bruker Daltonik GmbH, Bremen, Germany) in positive mode. Silica gel 60 (0.063–0.200 mesh, Merck, Whitehouse Station, NJ, USA) was used for column chromatography, and precoated silica gel plates (Merck 60 F254 0.2 mm) were used for thin layer chromatography (TLC). Monitoring of the reaction progress and product purification was carried out by TLC.

#### 2.1.2. General Procedure for the Synthesis of Bromoalkyl Derivatives of 4-Hydroxyacetophenone (**2a**–**h**)

These compounds were obtained as described below. A mixture of 4-hydroxyacetophenone (1 mmol), K_2_CO_3_ (1.5 mmol), and DMF (10 mL) was placed in a flat-bottomed flask of 25 mL equipped with a magnetic stirring bar and stirred for a period of 30 min. 1,ω-dibromoalkane (1.2 mmol) was added to the reaction, and the mixture was sonicated for 1 h at 25 °C. After this time, water was added, and the mixture was transferred to a separation funnel and extracted with ethyl acetate. Anhydrous sodium sulfate was used to dry the organic phase. The liquid phase was concentrated under reduced pressure on a rotatory evaporator, and the residue was purified by flash chromatography on silica gel using a mixture of hexanes/ethyl acetate of different ratios as eluent. Bromoalkyl derivatives were obtained in yields ranging from 73–84%.

1-(4-(2-bromoethoxy)phenyl)ethan-1-one (**2a**): Yield 73%, white solid; m.p. 61–63 °C; ^1^H NMR (300 MHz, Chloroform-d) δ 7.98 (d, *J* = 8.9 Hz, 2H), 6.99 (d, *J* = 8.9 Hz, 2H), 4.39 (t, *J* = 6.2 Hz, 2H), 3.70 (t, *J* = 6.2 Hz, 2H), 2.60 (s, 3H). ^13^C NMR (75 MHz, Chloroform-d) δ 196.85, 161.97, 130.89, 130.72 (2C), 114.32 (2C), 67.87, 28.71, 26.47.

1-(4-(3-bromopropoxy)phenyl)ethan-1-one (**2b**): Yield 83%, white oil; ^1^H NMR (300 MHz, Chloroform-d) δ 7.98 (d, *J* = 8.9 Hz, 2H), 6.98 (d, *J* = 8.9 Hz, 2H), 4.22 (t, *J* = 6.4 Hz, 2H), 3.65 (t, *J* = 5.8 Hz, 1H), 2.60 (s, 3H), 2.44-2-32 (m, 2H). ^13^C NMR (75 MHz, Chloroform-d) δ 196.85, 162.59, 130.66 (2C), 130.61, 114.17 (2C), 65.50, 32.13, 29.79, 26.43.

1-(4-(4-bromobutoxy)phenyl)ethan-1-one (**2c**): Yield 81%, white oil; ^1^H NMR (300 MHz, Chloroform-d) δ 7.96 (d, *J* = 8.9 Hz, 2H), 6.95 (d, *J* = 8.9 Hz, 2H), 4.10 (t, *J* = 5.9 Hz, 2H), 3.53 (t, *J* = 6.4 Hz, 2H), 2.59 (s, 3H), 2.17–2.06 (m, 2H), 2.06–1.95 (m, 2H). ^13^C NMR (75 MHz, Chloroform-d) δ 196.86, 162.79, 130.64 (2C), 130.32, 114.12 (2C), 67.11, 33.38, 29.36, 27.76, 26.42.

1-(4-((5-bromopentyl)oxy)phenyl)ethan-1-one (**2d**): Yield 84%, white oil; ^1^H NMR (300 MHz, Chloroform-d) δ 7.97 (d, *J* = 8.9 Hz, 2H), 6.96 (d, *J* = 8.9 Hz, 2H), 4.06 (t, *J* = 6.4 Hz, 2H), 3.47 (t, *J* = 6.7 Hz, 2H), 2.60 (s, 3H), 2.00–1.90 (m, 2H), 1.90–1.78 (m, 4H). ^13^C NMR (75 MHz, Chloroform-d) δ 196.89, 163.02, 130.63 (2C), 130.17, 114.13 (2C), 67.99, 32.65, 28.96, 27.91, 26.41, 25.27.

1-(4-((6-bromohexyl)oxy)phenyl)ethan-1-one (**2e**): Yield 86%, white solid; m.p. 42–46 °C; ^1^H NMR (300 MHz, Chloroform-d) δ 7.96 (d, *J* = 8.9 Hz, 2H), 6.95 (d, *J* = 8.9 Hz, 2H), 4.06 (t, *J* = 6.4 Hz, 2H), 3.47 (t, *J* = 6.7 Hz, 2H), 2.59 (s, 3H), 2.00–1.90 (m, 2H), 1.90–1.79 (m, 2H), 1.61–1.49 (m, 4H). ^13^C NMR (75 MHz, Chloroform-d) δ 196.86, 163.02, 130.63 (2C), 130.17, 114.13 (2C), 67.99, 32.65, 31.00, 28.96, 27.91, 26.41, 25.27.

1-(4-((8-bromooctyl)oxy)phenyl)ethan-1-one (**2f**): Yield 78%, white solid; m.p. 48–50 °C; ^1^H NMR (300 MHz, Chloroform-d) δ 7.96 (d, *J* = 8.9 Hz, 2H), 6.96 (d, *J* = 8.9 Hz, 2H), 4.05 (t, *J* = 6.5 Hz, 2H), 3.45 (t, *J* = 6.8 Hz, 2H), 2.59 (s, 3H), 1.97–1.76 (m, 4H), 1.57–1.33 (m, 8H). ^13^C NMR (75 MHz, Chloroform-d) δ 196.87, 163.10, 130.62 (2C), 130.11, 114.14 (2C), 68.18, 34.07, 32.78, 29.18, 29.08, 28.70, 28.10, 26.41, 25.92.

1-(4-((9-bromononyl)oxy)phenyl)ethan-1-one (**2g**): Yield 75%, white solid; m.p. 50–52 °C; ^1^H NMR (300 MHz, Chloroform-d) δ 7.96 (d, *J* = 8.8 Hz, 2H), 6.95 (m, *J* = 8.8 Hz, 2H), 4.05 (t, *J* = 6.5 Hz, 2H), 3.45 (t, *J* = 6.8 Hz, 2H), 2.59 (s, 3H), 1.95–178 (m, 4H), 1.57–1.33 (m, 10H). ^13^C NMR (75 MHz, Chloroform-d) δ 196.87, 163.11, 130.62 (2C), 130.09, 114.14 (2C), 68.22, 34.11, 32.81, 29.36, 29.25, 29.10, 28.71, 28.15, 26.40, 25.97.

1-(4-((12-bromododecyl)oxy)phenyl)ethan-1-one (**2h**): Yield 79%, white solid; m.p. 39–40 °C; ^1^H NMR (300 MHz, Chloroform-d) δ 7.97 (d, *J* = 8.8 Hz, 2H), 6.96 (d, *J* = 8.8 Hz, 2H), 4.06 (t, *J* = 6.5 Hz, 2H), 3.45 (t, *J* = 6.9 Hz, 2H), 2.60 (s, 3H), 1.96–1.78 (m, 4H), 1.56–1.26 (m, 16H). ^13^C NMR (75 MHz, Chloroform-d) δ 196.89, 163.14, 130.62 (2C), 130.09, 114.14 (2C), 68.28, 34.16, 32.86, 29.55 (3C), 29.46, 29.38, 29.13, 28.80, 28.20, 26.40, 26.00.

#### 2.1.3. General Procedure for the Synthesis of Alkylazides Derivatives of the 4-Hydroxyacetophenone (**3a**–**h**)

Compounds **2a**–**h** (1 mmol), sodium azide (3 mmol), and DMF (5 mL) were placed in a 10 mL flat-bottomed flask equipped with a magnetic stirring bar. Then, the mixture was heated to 100 °C at 200 W for a period of 15 min under microwave irradiation. Then, water was added, and the mixture was transferred to a separation funnel and extracted with ethyl acetate. Anhydrous sodium sulfate was used to dry the organic phase. The liquid phase was concentrated under reduced pressure on a rotatory evaporator, and the residue was purified by flash chromatography on silica gel using a mixture of hexanes/ethyl acetate of different ratios as the eluent. Alkylazide derivatives were obtained in yields ranging from 85–96%.

1-(4-(2-azidoethoxy)phenyl)ethan-1-one (**3a**): Yield 88%, yellow oil; ^1^H NMR (300 MHz, Chloroform-d) δ 7.97 (d, *J* = 8.9 Hz, 2H), 6.98 (d, *J* = 8.9 Hz, 2H), 3.66 (t, *J* = 4.9 Hz, 2H), 4.23 (t, *J* = 4.9 Hz, 2H), 2.58 (s, 3H). ^13^C NMR (75 MHz, Chloroform-d) δ 196.81 (C=O), 162.05, 130.84, 130.66 (2C), 114.22 (2C), 67.10, 50.03, 26.43.

1-(4-(3-azidopropoxy)phenyl)ethan-1-one (**3b**): Yield 93%, yellow oil; ^1^H NMR (300 MHz, Chloroform-d) δ 7.98 (d, *J* = 8.9 Hz, 2H), 6.98 (d, *J* = 8.9 Hz, 2H), 4.17 (t, *J* = 5.9 Hz, 2H), 3.58 (t, *J* = 6.6 Hz, 2H), 2.61 (s, 3H), 2.18–2.08 (m, 2H). ^13^C NMR (75 MHz, Chloroform-d) δ 196.89 (C=O), 162.58, 130.67 (2C), 130.51, 114.15 (2C), 64.72, 48.10, 28.67, 26.43.

1-(4-(4-azidobutoxy)phenyl)ethan-1-one (**3c**): Yield 92%, yellow oil; ^1^H NMR (300 MHz, Chloroform-d) δ 7.97 (d, *J* = 8.9 Hz, 2H), 6.96 (d, *J* = 8.9 Hz, 2H), 4.10 (t, *J* = 5.9 Hz, 2H), 3.42 (t, *J* = 6.6 Hz, 2H), 2.60 (s, 3H), 2.01–1.89 (m, 2H), 1.89–1.76 (m, 2H). ^13^C NMR (75 MHz, Chloroform-d) δ 196.87 (C=O), 162.79, 130.65 (2C), 130.33, 114.11 (2C), 67.43, 51.14, 26.41, 25.70.

1-(4-((5-azidopentyl)oxy)phenyl)ethan-1-one (**3d**): Yield 96%, yellow oil; ^1^H NMR (300 MHz, Chloroform-d) δ 7.96 (d, *J* = 8.8 Hz, 2H), 6.95 (d, *J* = 8.9 Hz, 2H), 4.06 (t, *J* = 6.3 Hz, 2H), 3.35 (t, *J* = 6.6 Hz, 2H), 2.58 (s, 3H), 1.93–1.81 (m, 2H), 1.77–1.66 (m, 2H), 1.66–1.54 (m, 2H). ^13^C NMR (75 MHz, Chloroform-d) δ 196.89 (C=O), 162.94, 130.63 (2C), 130.21, 114.12 (2C), 67.83, 51.32, 28.68, 28.64, 26.39, 23.36.

1-(4-((6-azidohexyl)oxy)phenyl)ethan-1-one (**3e**): Yield 85%, yellow oil; ^1^H NMR (300 MHz, Chloroform-d) δ 7.95 (d, *J* = 8.9 Hz, 2H), 6.94 (d, *J* = 8.9 Hz, 2H), 4.05 (t, *J* = 6.4 Hz, 2H), 3.31 (t, *J* = 6.8 Hz, 2H), 2.58 (s, 3H), 1.91–1.78 (m, 2H), 1.72–1.60 (m, 2H), 1.59–1.41 (m, 4H). ^13^C NMR (75 MHz, Chloroform-d) δ 196.87 (C=O), 162.59, 130.61 (2C), 130.15, 114.11 (2C), 67.97, 51.36, 28.98, 28.79, 26.49, 26.38, 25.64.

1-(4-((8-azidooctyl)oxy)phenyl)ethan-1-one (**3f**): Yield 89%, yellow oil; ^1^H NMR (300 MHz, Chloroform-d) δ 7.97 (d, *J* = 8.9 Hz, 2H), 6.96 (d, *J* = 8.9 Hz, 2H), 4.06 (t, *J* = 6.5 Hz, 2H), 3.31 (t, *J* = 6.9 Hz, 2H), 2.60 (s, 3H), 1.91-1.78 (m, 2H), 1.71–1.58 (m, 2H), 1.58–1.35 (m, 8H). ^13^C NMR (75 MHz, Chloroform-d) δ 196.90 (C=O), 163.10, 130.62 (2C), 130.12, 114.13 (2C), 68.18, 51.47, 29.22, 29.09, 28.84, 26.67, 26.40, 25.92.

1-(4-((9-azidononyl)oxy)phenyl)ethan-1-one (**3g**): Yield 87%, yellow oil; ^1^H NMR (300 MHz, Chloroform-d) δ 7.97 (d, *J* = 8.8 Hz, 2H), 6.96 (d, *J* = 8.8 Hz, 2H), 4.06 (t, *J* = 6.5 Hz, 2H), 3.30 (t, *J* = 6.9 Hz, 2H), 2.60 (s, 3H), 1.91–1.78 (m, 2H), 1.71–1.57 (m, 2H), 1.58–1.33 (m, 10H). ^13^C NMR (75 MHz, Chloroform-d) δ 196.89 (C=O), 163.12, 130.62 (2C), 130.11, 114.14 (2C), 68.22, 51.48, 29.41, 29.26, 29.10, 28.85, 26.72, 26.40, 25.97.

1-(4-((12-azidododecyl)oxy)phenyl)ethan-1-one (**3h**): Yield 88%, yellow solid; m.p. 38–40 °C; ^1^H NMR (300 MHz, Chloroform-d) δ 7.97 (d, *J* = 8.9 Hz, 2H), 6.96 (d, *J* = 8.9 Hz, 2H), 4.06 (t, *J* = 6.5 Hz, 2H), 3.30 (t, *J* = 6.9 Hz, 2H), 2.60 (s, 3H), 1.94–1.78 (m, 2H), 1.70–1.58 (m, 2H), 1.56–1.25 (m, 16H). ^13^C NMR (75 MHz, Chloroform-d) δ 196.91 (C=O), 163.15, 130.62 (2C), 130.08, 114.14 (2C), 68.28, 51.51, 29.56 (2C), 29.50, 29.38, 29.18, 29.12, 28.86, 26.74, 26.39, 26.00.

#### 2.1.4. General Procedure for the Synthesis of Alkylazide Derivatives of the (*E*)-3-(4-Hydroxy-3-methoxyphenyl)-1-(4-hydroxyphenyl)prop-2-en-1-one (**4a**–**h**)

Acetic acid (5 mmol) was added to pyrrolidine (5 mmol) cooled in a bath of ice and water for 10 min, and then the mixture was warmed to 30 °C. Then, compounds **3a**–**h** (1 mmol) and vanillin (1 mmol) were added. The mixture was stirred at 30 °C for 2 h. Then, water was added, and the mixture was transferred to a separation funnel and extracted with ethyl acetate. Anhydrous sodium sulfate was used to dry the organic phase. The liquid phase was concentrated under reduced pressure on a rotatory evaporator, and the residue was purified by flash chromatography on silica gel using a mixture of hexanes/ethyl acetate of different ratios as the eluent. Alkylazide derivatives were obtained in yields ranging from 68–82%.

(*E*)-1-(4-(2-azidoethoxy)phenyl)-3-(4-hydroxy-3-methoxyphenyl)prop-2-en-1-one (**4a**): Yield 82%, yellow solid; m.p. 94–95 °C; ^1^H NMR (600 MHz, Chloroform-d) δ 8.03 (d, *J* = 8.8 Hz, 1H), 7.74 (d, *J* = 15.5 Hz, 1H), 7.38 (d, *J* = 15.5 Hz, 1H), 7.21 (dd, *J* = 8.2, 1.8 Hz, 1H), 7.12 (d, *J* = 1.8 Hz, 1H), 6.99 (d, *J* = 8.8 Hz, 1H), 6.95 (d, *J* = 8.2 Hz, 1H), 6.09 (s, 1H), 4.22 (t, *J* = 5.0 Hz, 2H), 3.64 (t, *J* = 5.0 Hz, 2H). 13C NMR (75 MHz, Chloroform-d) δ 188.87, 161.84, 148.21, 146.83, 144.65, 131.91, 130.80 (2C), 127.60, 123.26, 119.42, 114.90, 114.33 (2C), 110.07, 67.12, 56.06, 50.07.

(*E*)-1-(4-(3-azidopropoxy)phenyl)-3-(4-hydroxy-3-methoxyphenyl)prop-2-en-1-one (**4b**): Yield 76%, yellow solid; m.p. 91–92 °C; ^1^H NMR (300 MHz, Chloroform-d) δ 8.07 (d, *J* = 8.6 Hz, 2H), 7.79 (d, *J* = 15.5 Hz, 1H), 7.43 (d, *J* = 15.5 Hz, 1H), 7.25 (d, *J* = 8.2 Hz, 1H), 7.17 (sapp, 1H), 7.01 (d, *J* = 8.6 Hz, 2H), 7.00 (d, *J* = 8.2 Hz, 2H), 4.17 (t, *J* = 5.9 Hz, 2H), 3.99 (s, 3H), 3.58 (t, *J* = 6.5 Hz, 2H), 2.19-2.06 (m, 2H). ^13^C NMR (75 MHz, Chloroform-d) δ 188.87, 162.38, 148.22, 146.87, 144.53, 131.48, 130.79 (2C), 127.61, 123.23, 119.42, 114.92 (2C), 114.26, 110.10, 64.75, 56.04, 48.13, 28.68.

(*E*)-1-(4-(4-azidobutoxy)phenyl)-3-(4-hydroxy-3-methoxyphenyl)prop-2-en-1-one (**4c**): Yield 77%, yellow solid; m.p. 63–65 °C; ^1^H NMR (300 MHz, Chloroform-d) δ 8.07 (d, *J* = 8.7 Hz, 2H), 7.79 (d, *J* = 15.5 Hz, 1H), 7.46 (s, 1H), 7.26 (dd, *J* = 8.2, 1.3 Hz, 1H), 7.17 (s, 1H), 7.00 (m, 3H), 4.11 (t, *J* = 5.9 Hz, 2H), 4.00 (s, 3H), 3.43 (t, *J* = 6.5 Hz, 2H), 2.03–1.76 (m, 4H). ^13^C NMR (75 MHz, Chloroform-d) δ 188.85, 162.61, 148.18, 146.84, 144.45, 131.32, 130.79 (2C), 127.64, 123.21, 119.46, 114.90, 114.23 (2C), 110.08, 67.45, 56.05, 51.16, 26.42, 25.72.

(*E*)-1-(4-((5-azidopentyl)oxy)phenyl)-3-(4-hydroxy-3-methoxyphenyl)prop-2-en-1-one (**4d**): Yield 86%, yellow solid; m.p. 77–79 °C; 1H NMR (300 MHz, Chloroform-d) δ 8.07 (d, *J* = 8.8 Hz, 2H), 7.79 (d, *J* = 15.5 Hz, 1H), 7.44 (d, *J* = 15.5 Hz, 1H), 7.26 (dd, *J* = 8.2, 1.7 Hz, 1H), 7.17 (d, *J* = 1.7 Hz, 1H), 7.00 (d, *J* = 8.8 Hz, 2H), 7.00 (d, *J* = 8.2 Hz, 1H), 4.09 (t, *J* = 6.3 Hz, 2H), 4.00 (s, 3H), 3.37 (t, *J* = 6.6 Hz, 2H), 1.96-1.84 (m, 2H), 1.82–1.55 (m, 4H). ^13^C NMR (75 MHz, Chloroform-d) δ 188.86, 162.75, 148.15, 146.83, 144.40, 131.22, 130.78 (2C), 127.66, 123.19, 119.50, 114.90, 114.24 (2C), 110.07, 67.85, 56.05, 51.35, 28.73, 28.67, 23.40.

(*E*)-1-(4-((6-azidohexyl)oxy)phenyl)-3-(4-hydroxy-3-methoxyphenyl)prop-2-en-1-one (**4e**): Yield 84%, yellow solid; m.p. 89–90 °C; ^1^H NMR (300 MHz, Chloroform-d) δ 8.07 (d, *J* = 8.8 Hz, 2H), 7.79 (d, *J* = 15.6 Hz, 1H), 7.44 (d, *J* = 15.6 Hz, 1H), 7.26 (dd, *J* = 8.2, 1.7 Hz, 1H), 7.17 (d, *J* = 1.7 Hz, 1H), 7.01 (d, *J* = 8.8 Hz, 2H), 7.00 (d, *J* = 8.2 Hz, 1H), 4.09 (t, *J* = 6.4 Hz, 2H), 4.00 (s, 3H), 3.34 (t, *J* = 6.8 Hz, 2H), 1.95–1.81 (m, 2H), 1.79–1.63 (m, 2H), 1.63–1.43 (m, 4H). ^13^C NMR (75 MHz, Chloroform-d) δ 188.85, 162.83, 148.13, 146.82, 144.36, 131.16, 130.77 (2C), 127.68, 123.18, 119.52, 114.88, 114.25 (2C), 110.06, 68.00, 56.05, 51.39, 29.03, 28.82, 26.53, 25.69.

(*E*)-1-(4-((8-azidooctyl)oxy)phenyl)-3-(4-hydroxy-3-methoxyphenyl)prop-2-en-1-one (**4f**): Yield 79%, yellow solid; m.p. 91–94 °C; ^1^H NMR (600 MHz, Chloroform-d) δ 8.03 (d, *J* = 8.8 Hz, 2H), 7.75 (d, *J* = 15.5 Hz, 1H), 7.41 (d, *J* = 15.5 Hz, 1H), 7.21 (dd, *J* = 8.2, 1.4 Hz, 1H), 7.13 (sapparent, 1H), 6.98–695 (m, 3H), 4.03 (t, *J* = 6.5 Hz, 3H), 3.94 (s, 3H), 3.27 (t, *J* = 6.9 Hz, 3H), 1.85–1.77 (m, 2H), 1.64–1.58 (m, 2H), 1.52–1.44 (m, 2H), 1.43–1.33 (m, 6H). ^13^C NMR (75 MHz, Chloroform-d) δ 191.00, 162.93, 148.16, 146.84, 144.37, 131.08, 130.77 (2C), 127.67, 123.19, 119.50, 114.90, 114.26 (2C), 110.08, 68.21, 56.14, 56.04, 51.48, 29.24, 29.10, 28.84, 26.68, 25.94.

(*E*)-1-(4-((9-azidononyl)oxy)phenyl)-3-(4-hydroxy-3-methoxyphenyl)prop-2-en-1-one (**4g**): Yield 81%, yellow solid; m.p. 72–75 °C; ^1^H NMR (300 MHz, Chloroform-d) δ 8.07 (d, *J* = 8.8 Hz, 2H), 7.79 (d, *J* = 15.5 Hz, 1H), 7.44 (d, *J* = 15.5 Hz, 1H), 7.26 (dd, *J* = 8.2, 1.7 Hz, 1H), 7.17 (d, *J* = 1.7 Hz, 1H), 7.01 (d, *J* = 8.8 Hz, 2H), 7.00 (d, *J* = 8.2 Hz, 1H), 4.08 (t, *J* = 6.5 Hz, 2H), 4.00 (s, 3H), 3.31 (t, *J* = 6.9 Hz, 2H), 1.93–1.80 (m, 2H), 1.71–1.58 (m, 2H), 1.57–1.31 (m, 10H). ^13^C NMR (75 MHz, Chloroform-d) δ 188.86, 162.94, 148.12, 146.81, 144.33, 131.08, 130.76 (2C), 127.69, 123.18, 119.53, 114.88, 114.26 (2C), 110.06, 68.24, 56.15, 56.05, 51.49, 29.42, 29.28, 29.11, 28.86, 26.72, 25.99.

(*E*)-1-(4-((12-azidododecyl)oxy)phenyl)-3-(4-hydroxy-3-methoxyphenyl)prop-2-en-1-one (**4h**): Yield 68%, yellow solid; m.p. 66–69 °C; 1H NMR (300 MHz, Chloroform-d) δ 8.07 (d, *J* = 8.8 Hz, 2H), 7.79 (d, *J* = 15.5 Hz, 1H), 7.44 (d, *J* = 15.5 Hz, 1H), 7.27 (dd, *J* = 8.2, 1.4 Hz, 1H), 7.18 (d, *J* = 1.4 Hz, 1H), 7.01 (d, *J* = 8.8 Hz, 2H), 7.00 (d, *J* = 8.2 Hz, 1H), 4.08 (t, *J* = 6.5 Hz, 2H), 3.30 (t, *J* = 6.9 Hz, 2H), 1.92–1.78 (m, 2H), 1.75–1.58 (m, 2H), 1.56–1.25 (m, 16H). ^13^C NMR (75 MHz, Chloroform-d) δ 188.85, 162.96, 148.09, 146.79, 144.30, 131.07, 130.76 (2C), 127.71, 123.17, 119.57, 114.86, 114.27 (2C), 110.04, 68.29, 56.04, 51.51, 29.74, 29.56 (2C), 29.51, 29.39, 29.19, 28.87, 26.75, 26.02.

#### 2.1.5. Synthesis of Propargyl-5-FU (5)

5-fluorouracil (1 mmol), HMDS (2.0 mmol), KI (0.5 mmol), and ammonium sulfate (0.5 mmol) were placed in a 25 mL flat-bottomed flask. The mixture was refluxed at 120 °C for 3 h. Then, the temperature was lowered to 90 °C, and 5 mL of acetonitrile and propargyl bromide (2.0 mmol) was added; the mixture was left to reflux at 90 °C for 12 h. The reaction was then concentrated under reduced pressure on a rotatory evaporator, and the residue was purified by flash chromatography on silica gel using a mixture of hexanes/ethyl acetate of different ratios as the eluent to obtain compounds **5** in 70% yield.

Yield 70%, solid white, m.p. 169–171 °C; 1H NMR (300 MHz, DMSO-d6) δ 11.93 (s, 1H), 8.13 (d, *J* = 6.6 Hz, 1H), 4.46 (d, *J* = 2.5 Hz, 2H), 3.45 (t, *J* = 2.5 Hz, 1H). 13C NMR (75 MHz, DMSO-d6) δ 157.98 and 157.63 (F-C-C=O), 149.51 (HN-C=O-N), 141.76 and 138.71 (C-F), 129.64 and 129.19 (F-C=CH-N), 78.60, 76.59, 37.45 (-CH2-N).

#### 2.1.6. General Procedure for the Synthesis of 5-FU-Curcumin Hybrids (**6a**–**h**)

In a 10 mL flat-bottomed flask, were placed propargyl-5-FU (**5**) (1 mmol), chalcone-alkylazides (**4a**–**h**) (1 mmol) DMF (5 mL), and the mixture was sonicated for 5 min to 40 °C. After this time, was added a mixture of ascorbic acid (0.5 mmol), copper acetate (0.5 mmol), DMF (1 mL), and water (1 mL) and the reaction mixture and sonicated for 1 h to 40 °C. Then, 10% HCl was added and extracted with ethyl acetate. The organic phase was dried on anhydrous sodium sulfate, filtered, and concentrated under reduced pressure, and the residue was subjected to crystallization (MeOH:H_2_O, 1:1 ratio). Finally, the solid obtained was purified by preparative chromatography on silica gel to obtain compounds **6a**–**h** (88–96% yield).

(*E*)-5-fluoro-1-((1-(2-(4-(3-(4-hydroxy-3-methoxyphenyl)acryloyl)phenoxy)ethyl)-1H-1,2,3-triazol-4-yl)methyl)pyrimidine-2,4(1H,3H)-dione (**6a**): Yield 92%, solid yellow, m.p. 124–129 °C; ^1^H NMR (600 MHz, DMSO-d6) δ 8.22 (s, 1H), 8.19 (d, *J* = 7.0 Hz, 1H), 8.13 (d, *J* = 8.7 Hz, 2H), 7.74 (d, *J* = 15.4 Hz, 1H), 7.65 (d, *J* = 15.4 Hz, 1H), 7.50 (s, 1H), 7.27 (d, *J* = 8.1 Hz, 1H), 7.06 (d, *J* = 8.7 Hz, 2H), 6.84 (d, *J* = 8.1 Hz, 1H), 4.92 (s, 1H), 4.81 (t, *J* = 4.8 Hz, 2H), 4.53 (t, *J* = 4.9 Hz, 2H), 3.86 (s, 3H). ^13^C NMR (151 MHz, DMSO-d6) δ 187.71 (C=O), 162.16 (N-C=O), 161.96 (Ar-O), 157.99 and 157.81 (F-C-C=O), 150.02 (Ar-O), 149.84 (Ar-O), 148.43 (C=C), 144.67 (triazolyl), 140.88 and 139.35 (F-C), 131.74, 131.17 (2C-Ar), 130.50 and 130.27 (CH-C-F), 130.19, 126.84, 124.79, 124.43 (triazolyl), 119.03, 116.04, 114.96 (2C-Ar), 114.87, 112.13, 66.86, 56.31 (OMe), 49.47, 43.22. HRMS (ESI), calcd for C_25_H_22_FN_5_O_6_ [M+H]^+^: 508.1626, found: 508.1628.

(*E*)-5-fluoro-1-((1-(3-(4-(3-(4-hydroxy-3-methoxyphenyl)acryloyl)phenoxy)propyl)-1H-1,2,3-triazol-4-yl)methyl)pyrimidine-2,4(1H,3H)-dione (**6b**): Yield 92%, solid yellow, m.p. 124–129 °C; ^1^H NMR (600 MHz, DMSO-d6) δ 8.21 (s, 1H), 8.17 (d, *J* = 5.3, 1H), 8.11 (d, *J* = 8.0 Hz, 2H), 7.73 (d, *J* = 15.4 Hz, 1H), 7.63 (d, *J* = 15.4 Hz, 1H), 7.49 (s, 1H), 7.25 (d, *J* = 7.8 Hz, 1H), 7.01 (d, *J* = 8.0 Hz, 2H), 6.82 (d, *J* = 7.8 Hz, 1H), 4.90 (s, 2H), 4.54 (t, *J* = 5.8 Hz, 2H), 4.10 (t, *J* = 5.2 Hz, 2H), 3.87 (s, 3H), 3.52–3.28 (m, 2H). ^13^C NMR (151 MHz, DMSO-d6) δ 187.69 (C=O), 162.79 (N-C=O), 162.47 (Ar-O), 158.00 and 157.82 (F-C-C=O), 149.98 (Ar-O), 149.83 (Ar-O), 148.42 (C=C), 144.52 (triazolyl), 140.87 and 139.35 (F-C), 131.38, 131.15 (2C-Ar), 130.50 and 130.28 (CH-C-F), 126.85, 124.41 (triazolyl), 119.07, 116.02, 114.77 (2C-Ar), 112.08, 65.53, 56.31 (OMe), 47.27, 43.23, 29.71. HRMS (ESI), calcd for C_26_H_24_FN_5_O_6_ [M+H]^+^: 522.1779, found: 522.1783.

(*E*)-5-fluoro-1-((1-(4-(4-(3-(4-hydroxy-3-methoxyphenyl)acryloyl)phenoxy)butyl)-1H-1,2,3-triazol-4-yl)methyl)pyrimidine-2,4(1H,3H)-dione (**6c**): Yield 86%, solid yellow, m.p. 61–65 °C; ^1^H NMR (600 MHz, DMSO-d6) δ 8.21 (s, 1H), 8.17 (d, *J* = 5.0, 1H), 8.12 (d, *J* = 8.0 Hz, 2H), 7.74 (d, *J* = 15.4 Hz, 1H), 7.63 (d, *J* = 15.4 Hz, 1H), 7.48 (s, 1H), 7.25 (d, *J* = 7.8 Hz, 1H), 7.04 (d, *J* = 8.1 Hz, 2H), 6.84 (d, *J* = 7.8 Hz, 1H), 4.90 (s, 2H), 4.47–4.38 (m, 2H), 4.12–4.04 (m, 2H), 3.86 (s, 3H), 2.03–1.93 (m, 2H), 1.76–1.66 (m, 2H). ^13^C NMR (151 MHz, DMSO-d6) δ 187.69 (C=O), 162.81 (N-C=O), 162.72 (Ar-O), 158.00 and 157.82 (F-C-C=O), 149.99 (Ar-O), 149.83 (Ar-O), 148.42 (C=C), 144.50 (triazolyl), 140.87 and 139.35 (F-C), 131.20 (3C-Ar), 130.58 and 130.35 (CH-C-F), 126.84, 124.41 (triazolyl), 119.11, 116.05, 114.81 (2C-Ar), 112.14, 67.71, 56.38 (OMe), 49.76, 43.31, 26.91, 26.11. HRMS (ESI), calcd for C_27_H_26_FN_5_O_6_ [M+H]^+^: 536.1892, found: 536.1893.

(*E*)-5-fluoro-1-((1-(5-(4-(3-(4-hydroxy-3-methoxyphenyl)acryloyl)phenoxy)pentyl)-1H-1,2,3-triazol-4-yl)methyl)pyrimidine-2,4(1H,3H)-dione (**6d**): Yield 91%, solid yellow, m.p. 104–107 °C; ^1^H NMR (600 MHz, DMSO-d6) δ 8.18 (d, *J* = 6.6 Hz, 1H), 8.15–8.11 (m, 3H), 7.76 (d, *J* = 15.4 Hz, 1H), 7.65 (d, *J* = 15.4 Hz, 1H), 7.51 (s, 1H), 7.27 (d, *J* = 7.7 Hz, 1H), 7.05 (d, *J* = 8.7 Hz, 2H), 6.84 (d, *J* = 8.1 Hz, 1H), 4.91 (s, 2H), 4.38 (t, *J* = 7.0 Hz, 2H), 4.06 (t, *J* = 6.3 Hz, 2H), 3.88 (s, 3H), 1.93–1.85 (m, 2H), 1.81–1.73 (m, 2H), 1.45–1.36 (m, 2H). ^13^C NMR (151 MHz, DMSO-d6) δ 187.66 (C=O), 162.82 (N-C=O and Ar-O), 157.99 and 157.81 (F-C-C=O), 149.97 (Ar-O), 149.82 (Ar-O), 148.42 (C=C), 144.46 (triazolyl), 140.86 and 139.35 (F-C), 131.19 (2C-Ar), 131.13, 130.52 and 130.29 (CH-C-F), 126.86, 124.39 (triazolyl), 119.08, 116.03, 114.77 (2C-Ar), 112.10, 68.08, 56.33 (OMe), 49.85, 43.32, 29.80, 28.35, 22.95. HRMS (ESI), calcd for C_28_H_28_FN_5_O_6_ [M+H]^+^: 550.2062, found: 550.2064.

(*E*)-5-fluoro-1-((1-(6-(4-(3-(4-hydroxy-3-methoxyphenyl)acryloyl)phenoxy)hexyl)-1H-1,2,3-triazol-4-yl)methyl)pyrimidine-2,4(1H,3H)-dione (**6e**): Yield 94%, solid yellow, m.p. 118–124 °C; ^1^H NMR (600 MHz, DMSO-d6) δ 8.17 (d, *J* = 6.2 Hz, 1H), 8.15–8.11 (m, 3H), 7.75 (d, *J* = 15.4 Hz, 1H), 7.64 (d, *J* = 15.4 Hz, 1H), 7.50 (s, 1H), 7.26 (d, *J* = 8.0 Hz, 1H), 7.04 (d, *J* = 8.4 Hz, 2H), 6.84 (d, *J* = 8.0 Hz, 1H), 4.90 (s, 1H), 4.34 (t, *J* = 6.8 Hz, 2H), 4.04 (t, *J* = 6.1 Hz, 2H), 3.87 (s, 3H), 1.86–1.79 (m, 2H), 1.74–1.67 (m, 2H), 1.47–1.39 (m, 2H), 1.33–1.25 (m, 2H). ^13^C NMR (151 MHz, DMSO-d6) δ 187.67 (C=O), 162.87 (N-C=O and Ar-O), 157.99 and 157.82 (F-C-C=O), 149.97 (Ar-O), 149.83 (Ar-O), 148.43 (C=C), 144.45 (triazolyl), 140.88 and 139.36 (F-C), 131.19 (2C-Ar), 131.11, 130.49 and 130.26 (CH-C-F), 126.88, 124.37 (triazolyl), 119.08, 116.04, 114.75 (2C-Ar), 112.08, 68.19, 56.30 (OMe), 49.87, 43.29, 30.00, 28.80, 26.06, 25.32. HRMS (ESI), calcd for C_29_H_30_FN_5_O_6_ [M+H]^+^: 564.2257, found: 564.2262.

(*E*)-5-fluoro-1-((1-(8-(4-(3-(4-hydroxy-3-methoxyphenyl)acryloyl)phenoxy)octyl)-1H-1,2,3-triazol-4-yl)methyl)pyrimidine-2,4(1H,3H)-dione (**6f**): Yield 87%, solid yellow, m.p. 98–102 °C; ^1^H NMR (600 MHz, DMSO-d6) δ 8.20–8.09 (m, 4H), 7.74 (d, *J* = 15.4 Hz, 1H), 7.63 (d, *J* = 15.4 Hz, 1H), 7.49 (s, 1H), 7.25 (d, *J* = 7.7 Hz, 1H), 7.04 (d, *J* = 8.2 Hz, 2H), 6.83 (d, *J* = 8.2 Hz, 1H), 4.89 (s, 2H), 4.31 (t, *J* = 6.3 Hz, 2H), 4.04 (t, *J* = 6.0 Hz, 2H), 3.86 (s, 3H), 1.83–1.75 (m, 2H), 1.74–1.65 (m, 2H), 1.42–1.33 (m, 2H), 1.32–1.13 (m, 6H). ^13^C NMR (151 MHz, DMSO-d6) δ 187.65 (C=O), 162.90 (N-C=O and Ar-O), 157.98 and 157.80 (F-C-C=O), 149.96 (Ar-O), 149.82 (Ar-O), 148.42 (C=C), 144.44 (triazolyl), 140.87 and 139.35 (F-C), 131.18 (2C-Ar), 131.08, 130.71, 130.47 and 130.24 (CH-C-F), 130.11, 126.87, 124.36 (triazolyl), 119.07, 116.03, 114.75, 114.63 (2C-Ar), 112.08, 68.29, 56.31 (OMe), 49.93, 43.30, 30.04, 29.03, 28.95, 28.74, 26.24, 25.80. HRMS (ESI), calcd for C_31_H_34_FN_5_O_6_ [M+H]^+^: 592.2595, found: 592.2597.

(*E*)-5-fluoro-1-((1-(9-(4-(3-(4-hydroxy-3-methoxyphenyl)acryloyl)phenoxy)nonyl)-1H-1,2,3-triazol-4-yl)methyl)pyrimidine-2,4(1H,3H)-dione (**6g**): Yield 89%, solid yellow, m.p. 113–120 °C; ^1^H NMR (600 MHz, DMSO-d6) δ 8.17 (d, *J* = 5.6 Hz, 1H), 8.14 (s, 1H), 8.12 (d, 8.2 Hz, 2H), 7.73 (d, *J* = 15.4 Hz, 1H), 7.61 (d, *J* = 15.4 Hz, 1H), 7.47 (s, 1H), 7.24 (d, *J* = 8.0 Hz, 1H), 7.04 (d, *J* = 8.1 Hz, 2H), 6.85 (d, *J* = 8.0 Hz, 1H), 4.89 (s, 2H), 4.31 (t, *J* = 6.3 Hz, 4H), 4.04 (t, *J* = 6.0 Hz, 4H), 3.87 (s, 3H), 1.82–1.74 (m, 2H), 1.73–1.65 (m, 2H), 1.42–1.32 (m, 2H), 1.31–1.15 (m, 8H). ^13^C NMR (151 MHz, DMSO-d6) δ 187.67 (C=O), 162.90 (Ar-O), 162.83 (N-C=O), 157.98 and 157.80 (F-C-C=O), 150.03 (Ar-O), 149.81 (Ar-O), 148.43 (C=C), 144.46 (triazolyl), 140.84 and 139.32 (F-C), 131.23 (2C-Ar), 131.06, 130.58 and 130.35 (CH-C-F), 126.80, 124.41 (triazolyl), 119.11, 116.05, 114.80 (2C-Ar), 112.15, 68.40, 56.41 (OMe), 50.01, 43.26, 30.07, 29.24, 29.10, 29.00, 28.74, 26.28, 25.87. HRMS (ESI), calcd for C_32_H_36_FN_5_O_6_ [M+H]^+^: 606.2701, found: 606.2705.

(*E*)-5-fluoro-1-((1-(12-(4-(3-(4-hydroxy-3-methoxyphenyl)acryloyl)phenoxy)dodecyl)-1H-1,2,3-triazol-4-yl)methyl)pyrimidine-2,4(1H,3H)-dione (**6h**): Yield 88%, solid yellow, m.p. 129–135 °C; 1H NMR (600 MHz, DMSO-d6) δ 8.16–8.10 (m, 4H), 7.74 (d, *J* = 15.4 Hz, 1H), 7.63 (d, *J* = 15.4 Hz, 1H), 7.49 (s, 1H), 7.25 (d, *J* = 8.0 Hz, 1H), 7.04 (d, *J* = 8.2 Hz, 2H), 6.83 (d, *J* = 8.0 Hz, 1H), 4.89 (s, 2H), 4.31 (t, *J* = 6.3 Hz, 4H), 4.04 (t, *J* = 6.0 Hz, 3H), 3.86 (s, 3H), 1.83–1.75 (m, 2H), 1.74–1.65 (m, 2H), 1.42–1.33 (m, 2H), 1.32–1.17 (m, 14H). ^13^C NMR (151 MHz, DMSO-d6) δ 187.63 (C=O), 162.90 (Ar-O), 162.82 (N-C=O), 157.95 and 157.78 (F-C-C=O), 149.98 (Ar-O), 149.79 (Ar-O), 148.40 (C=C), 144.44 (triazolyl), 140.84 and 139.32 (F-C), 131.21 (2C-Ar), 131.05, 130.74, 130.55 and 130.33 (CH-C-F), 130.09, 129.92, 126.82, 124.41 (triazolyl), 119.12, 116.05, 114.79 (2C-Ar), 112.16, 68.42, 56.45 (OMe), 50.13, 35.01, 30.08, 29.45, 29.39, 29,37, 29.30, 29.21, 29.02, 28.81, 26.32, 25.91. HRMS (ESI), calcd for C_35_H_42_FN_5_O_6_ [M+H]^+^: 548.2779, found: 548.2782.

### 2.2. Biological Activity Assays

#### 2.2.1. Cell Lines and Culture Medium

SW480 (colon adenocarcinoma) and SW620 (derived metastatic) malignant cell lines, and the non-malignant lines HaCaT (human keratinocytes) and CHO-K1 (Chinese Hamster Ovary), were obtained from the European Collection of Authenticated Cell Cultures (ECACC, Porton Down, Salisbury, UK). The cells were maintained in Dulbecco’s Modified Eagle’s Medium (DMEM), supplemented with 10% equine serum previously inactivated at 56 °C, 1% penicillin/streptomycin, and 1% non-essential amino acids, and incubated at 37 °C in an atmosphere with 5% CO_2_. To carry out the experiments, the serum was diluted to 3% and additionally supplemented with 10 mg/mL insulin, 5 mg/mL transferrin, and 5 ng/mL selenium. Cells were used for experimentation when they reached 80–90% confluence, using a 1X trypsin enzymatic disintegration method. To avoid contamination by Mycoplasma spp., cell cultures were periodically analyzed by polymerase chain reaction (PCR) [22].

#### 2.2.2. Cell Viability

Cell viability was evaluated with a sulforhodamine B (SRB) assay. This technique provides an index of protein contents in living and adherent cells, whose optical density can be related to cell concentration. Initially, 20,000 cells were seeded per well in 96-well culture plates. An initial adherence period of 24 h was performed at 37 °C, 5% CO_2_, and after this, the medium was replaced by another that contained the hybrid compounds in different concentrations. Then, the cells were left incubating for 24 and 48 h under the same conditions previously described. After the respective incubation times, the cells were fixed with trichloroacetic acid (50% *v*/*v*) for 1 h at 4 °C and stained with 0.4% (*p*/*v*) of SRB. Subsequently, washings with 1% acetic acid were carried out to eliminate possible nonspecific bindings, and after solubilization with 10 mM Tris-base, the absorbance was read at 492 nm in a microplate reader (Mindray MR-96A) [23]. All the experiments were carried out in triplicate.

#### 2.2.3. Antiproliferative Activity

For this experiment, the same colorimetric method (SRB) from the evaluation of cell viability was used, although with some modifications. Initially, 2500 cells per well were seeded in 96-well culture plates, and a 24 h adherence period was performed at 37 °C in a 5% CO_2_ atmosphere. Afterwards, the cell lines were subjected to treatment with increasing concentrations (ranges dependent on the IC_50_ values) of the hybrid compounds that exhibited greater activity in the initial screening. For this assay, incubation periods of 0, 2, 4, 6, and 8 days were used, and the fixation, staining, and reading conditions were the same as those previously described for the cytotoxicity assay [23].

#### 2.2.4. Cell Cycle Analysis

The method used for the recognition of cell cycle phases (G0/G1, S, G2/M) was based on staining with a fluorescent dye (propidium iodide—PI), as a direct relationship between fluorescence intensity and DNA content can be established. For this method, 250,000 cells/well were initially seeded in 6-well culture plates with an adherence period of 24 h at 37 °C in a 5% CO_2_ atmosphere. After this time, the cells were treated with the respective IC_50_ value of the hybrid compound and vehicle control for 48 h. After treatment, cells were obtained by mechanical disaggregation, and the cell pellet was centrifuged and washed with a versene buffer. The cell pellet was fixed with 1.8 mL of cold 70% ethanol and kept at 4 °C overnight. Subsequently, two washes with a versene buffer were performed, and the pellet was resuspended in 300 µL of PBS containing 0.25 mg/mL of RNAsa and 0.1 mg/mL of PI. After a 30 min period of dark incubation, the fluorescence of 10,000 cells was analyzed using a FACS Canto II flow cytometer. The PI signal was analyzed with excitation at 488 nm using a sapphire laser, and fluorescence was detected at 610 nm. The data analysis was performed with FlowJo 10.8.1 software [24].

#### 2.2.5. Measurement of Mitochondrial Membrane Potential (∆Ψm)

The fluorescent dyes PI and DIOC6 (3,3’-dihexyloxacarbocyanine iodide) were used to observe the depolarization of the mitochondrial membrane as well as possible cell membrane damage. For this method, 250,000 cells/well were initially seeded in 6-well culture plates during a 24 h adherence period at 37 °C in a 5% CO_2_ atmosphere. After this time, the cells were treated with the respective IC_50_ of the hybrid compound and vehicle control for 48 h. After treatment, cells were obtained by mechanical disaggregation, stained with DiOC6 and PI, and incubated at room temperature for 30 min in the dark. Ten thousand events were analyzed by flow cytometry with excitation at 488 nm and emission detection with green (530/15 nm) and red (610/20 nm) filters [24].

#### 2.2.6. Cell Death Induction by 5-FU/Curcumin Hybrids

For this method, 250,000 cells/well were initially seeded in 6-well culture plates during a 24 h adherence period at 37 °C in a 5% CO_2_ atmosphere. After this time, the cells were treated with the respective IC_50_ of the hybrid compound and vehicle control for 48 h. After treatment, cells were obtained by mechanical disaggregation, resuspended in a solution with Annexin-V-FLUOS and PI, and incubated for 20 min in the dark before flow cytometry analysis [25].

#### 2.2.7. Determination of Apoptotic Biomarkers

To provide an approach to the possible molecular mechanisms through which the hybrid compounds induce an effect on the cell lines, different proteins related to the process of death by apoptosis were determined. For this method, 1,000,000 cells were initially seeded per Petri dish, with an adherence period of 24 h at 37 °C in a 5% CO_2_ atmosphere. After this time, the cells were treated with the respective IC_50_ of the hybrid compounds for 48 h. Later, the consumed medium was discarded, and after the addition of the versene buffer, the cells were obtained by mechanical disaggregation. The cells were then lysed with a cell lysis buffer and centrifuged to obtain the supernatant, which was used to determine the biomarkers of interest by enzyme-linked immunosorbent assays. These ELISA kits were provided by Elabscience Biotechnology Co., Houston, TX, USA [26].

#### 2.2.8. Statistical Analysis

The results were analyzed with GraphPad Prism version 5.00 for Windows (GraphPad Software, San Diego, CA, USA). The experiments were performed in triplicate, and the data were presented as the mean ± standard error (SE). Results were analyzed with one-way ANOVA, and statistical differences were identified using Tukey’s or Dunnett’s test. In all cases, a *p* < 0.05 was considered significant.

## 3. Results and Discussion

### 3.1. Chemistry

**Scheme 1:**
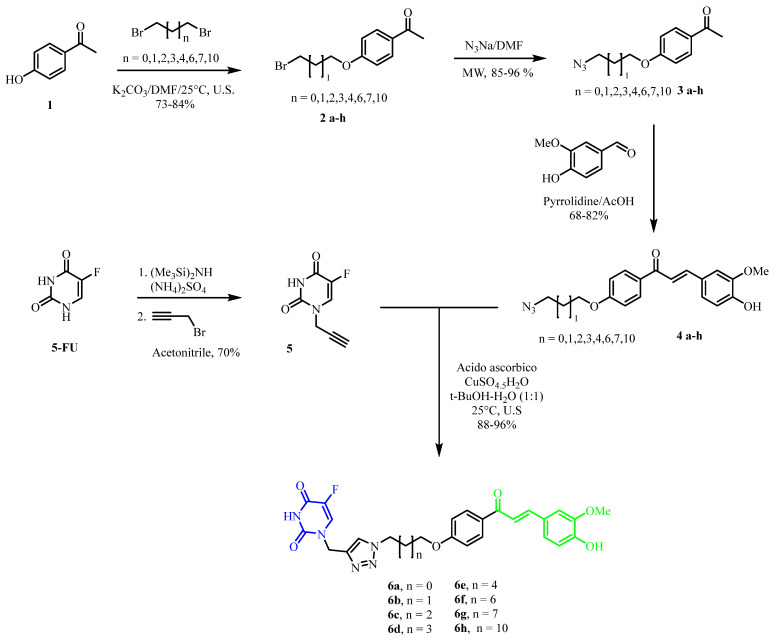
Synthesis of hybrids based on 5-FU and curcumin.

The synthesis of hybrids began with the Williamson reaction between acetophenone (1) and 1, ω-dibromoalkanes (ω = 3, 4, 5, 8, 9, and 12) to afford the bromo-alkylacetophenones **2a**–**h** with 73–84% yields [27,28]. Compounds **2a**–**h** were treated with sodium azide, leading to the obtention of the acetophenone-alkylazides **3a–h** in 85–96% yields [29,30]. The above compounds were subjected to an aldol condensation reaction with vanillin, giving the chalcones **4a**–**h** in 68–82% [31]. Besides, the reaction of 5-FU with propargyl bromide led to propargyl-5-FU (**5**) with a 70% yield [32]. Finally, the click reaction between chalcone-alkylazides **4a**–**h** and compound **5** led to the obtention of hybrids **6a**–**h** in 88–96% yields [30,33] (Scheme 1).

By combining the HRMS-ESI (*m*/*z*), 1H NMR, and 13C NMR spectra, the structures of all compounds were established. The characteristic [M+H]+ peaks corresponding to the molecular weights of the hybrids were shown by means of the HRMS-ESI (*m*/*z*) spectra. The assignments of all the signals to individual H or C-atoms have been performed based on typical δ-values and J-constants. The 1H NMR spectra of hybrids dissolved in DMSO showed around 8.17 ppm of a signal corresponding to 5-FU. 7.75 ppm and 7.65 ppm, respectively, a signal corresponding to a double bond of chalcone. 13C NMR spectra of hybrids exhibited a signal of around 187.66 ppm, corresponding to the carbonyl groups (C=O) of the chalcone. The triazolyl ring showed signals around 144.44 and 124.40 ppm. Finally, around 157.98 and 157.80 was observed the signal corresponding to F-C-C=O of 5-FU.

### 3.2. Biological Activity

#### 3.2.1. Effect of 5-FU/Curcumin Hybrids on SW480, SW620, HaCaT, and CHO-K1 Cell Viability

**Figure 3:**
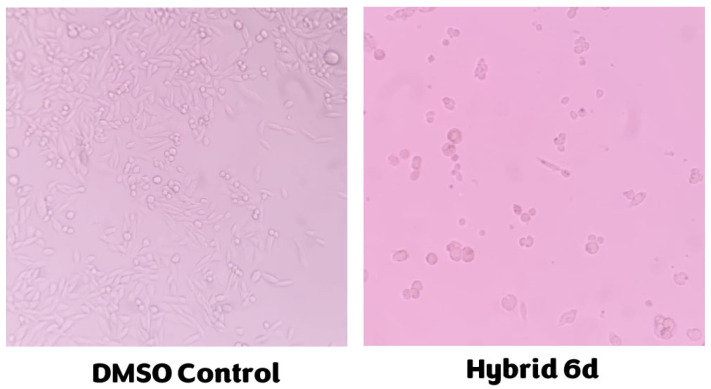
Representative images of SW480 cells 48 h after treatment with hybrid **6d**. Magnification: 20×.

The parental compounds curcumin and 5-fluorouracil (5-FU, the reference drug) were used as controls. All the compounds, with the exception of the 6g and 6h hybrids, showed an improvement in their cytotoxic activity against the SW480 and SW620 tumor cell lines 48 h after the start of the treatments, compared to the results obtained at 24 h (Table 1 and Table 2). The range of IC_50_ values was found between 2.43 and 29.71 µM in SW480 cells and between 7.51 and 45.10 µM in SW620 cells. These values were lower than those found for the starting compound curcumin and the reference drug 5-FU. Additionally, the hybrid compounds except for 6f, 6g, and 6h presented better IC_50_ values in the SW480 cell line regarding curcumin and the equimolar mixture. The same effect could be seen in SW620 cells with the hybrid compounds 6d and 6e. During the treatments, the cells were observed under an optical microscope. As shown in Figure 3, it was identified that cells presented an altered morphology with changes in size and shape, in addition to an evident decrease in cell population compared to the control. The severity of these changes increased as a function of time and dose.

The increase in cytotoxic activity over time was also reflected in some of the results obtained on the non-tumor cell lines HaCaT and CHO-K1, a situation that negatively affects the selectivity indices (SI) by generating a decrease in them. Only compounds **6a** and **6d** in SW480 cells and compounds **6d** and **6e** in SW620 cells increased their SI over 48 h post-treatment; moreover, these same compounds presented the lowest IC_50_ compared to the starting compounds and the equimolar mixture.

Similar results regarding the same starting compounds, curcumin and 5-FU, alone or in combination with other pharmacophores, have been reported. Zhang et al., 2019 [34] synthesized 8 hybrid compounds derived from the drugs 5-FU, cisplatin, and oxaliplatin and evaluated their cytotoxic and selective activity against human cancer cells: HeLa (cervical), MCF-7 (breast), CaCo-2 (colorectal), LoVo (colorectal), HCT116 (colon), A549 (lung), and non-tumor cells MRC5 (lung fibroblasts). They reported that, in general, all hybrids were more active than the drugs and their physical mixtures; furthermore, they were selective, with lower IC_50_ values in tumor cells than in human non-malignant lung cells. Similarly, Sharma et al. 2015 [35] synthesized 42 hybrid compounds derived from curcumin and isatin linked with a triazole and modified substituents on the aromatic ring. All compounds were evaluated against the human cancer cell lines THP-1 (leukemia), COLO-205 (colon), HCT-116 (colon), A549 (lung), HeLa (cervical), CAKI-I (kidney), PC-3 (prostate), and MiaPaCa-2 (pancreas), with the SA-2, SA-3, SA-4, and SA-7 hybrids showing the highest cytotoxic activity, and the SA-2 compound with a trimethoxyphenyl ring showing the best results (IC_50_ = 1.2 μM in HCT-116 cells).

**Figure 4:**
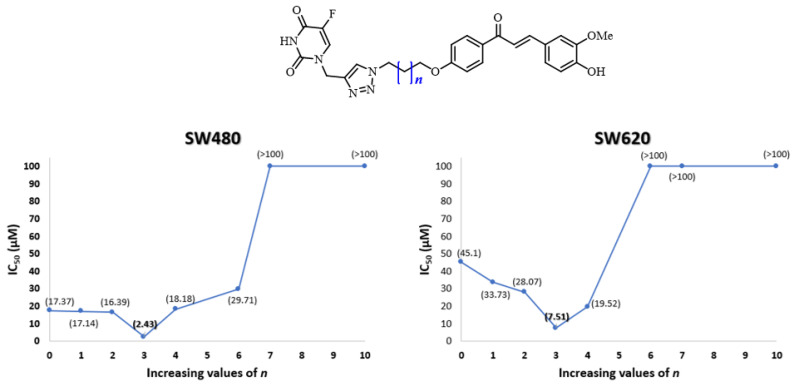
Variation of the treatment response curve with increasing carbon number. The values in parentheses correspond to the IC_50_ values of the hybrid compounds [36].

Regarding the structure–activity relationship (SAR), one of the objectives of the biological evaluation was to recognize the importance of the length of the aliphatic chain that links the chalcone analog of curcumin with the triazole, which in turn binds to 5-FU. After analyzing the results of the IC_50_ value, it was possible to evidence an improvement in the biological activity of the hybrid compounds when the chain length of the linker increased until it reached a length of 3 carbons. At this point, the most representative IC_50_ value of the entire screening was obtained (2.43 ± 0.33 µM in SW480 and 7.51 ± 1.47 µM in SW620). This allowed us to affirm that when the hybrid compound had a specific chain length of 3 carbons, the interaction with one or more molecular targets in the cell reached its maximum point of activity, and as a result, the highest levels of specific cytotoxicity were generated. Therefore, when observing the results obtained with the hybrids having chain lengths of 4, 6, 7, and 10 carbons, it was evident how the biological activity of these cell lines decreased (Figure 4).

In accordance with the cytotoxicity and high SI, compounds **6a**, **6d**, and **6e** were chosen to continue with the other biological assays.

#### 3.2.2. Antiproliferative Effect of 5-FU/Curcumin Hybrids on SW480 and SW620 Cells

**Figure 5:**
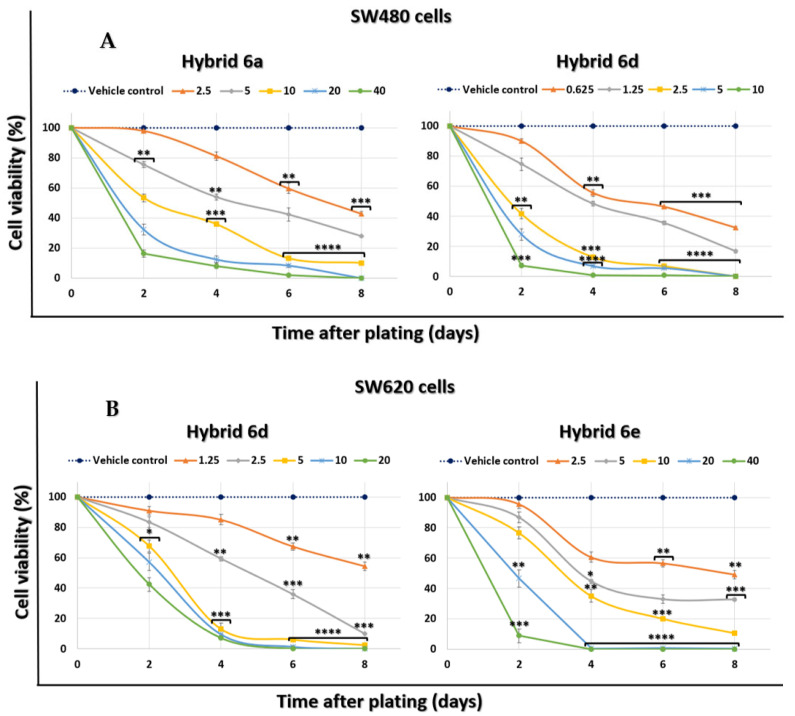
(**A**). Antiproliferative activity of hybrids **6a** and **6d** in SW480 cells; (**B**). Antiproliferative activity of hybrids **6d** and **6e** in SW620 cells. Data are presented as the mean ± SE of three independent experiments. (* *p* < 0.05; ** *p* < 0.01; *** *p* < 0.001; **** *p* < 0.0001). Vehicle control was assumed to be 100% viable.

According to the antiproliferative activity of compounds **6a** and **6d** (against the SW480 cell line) and **6d** and **6e** (against the SW620 cell line), it was possible to identify that the hybrid compounds showed a decrease in the percentages of cell viability with a dependence on concentration and time (Table 3). Thus, compound **6a** in the SW480 cell line showed significant differences in the inhibition percentages from day 2 at a concentration of 5 µM compared to the control and maintained a trend of decreasing viability until day 8 at all concentrations evaluated. A similar situation was presented with hybrid **6d** in this same cell line, where the main differences in inhibition began to appear from day 2 with concentrations higher than 2.5 µM (Figure 5).

Regarding the results obtained against the SW620 cell line with hybrids **6d** and **6e**, it was possible to state that from concentrations of 5 µM and 20 µM on day 2, a significant decrease in cell viability began to be observed, reaching inhibition percentages higher than 30%. Similar results were obtained and published by our research group from hybrids derived from 5-FU and genistein linked with a triazole [17], demonstrating the antiproliferative activity of compounds **4a** and **4g** on SW480 and SW620 cell lines with a significant inhibitory effect over time at low concentrations (6.25 μM). Similarly, Goldhahn et al., 2015 [37] evaluated the antiproliferative potential of the curcumin analog HP102 on Jurkat T cells, finding an enhanced effect with respect to the other analogs and curcumin.

#### 3.2.3. 5-FU/Curcumin Hybrids Induce Cell Cycle Arrest on SW480 and SW620 Cells

**Figure 6:**
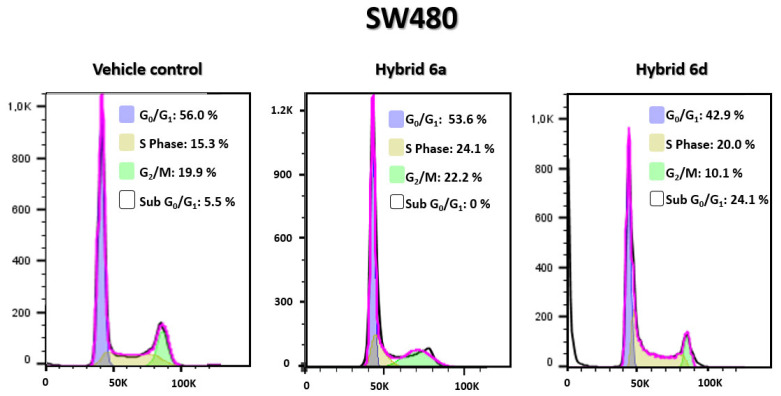

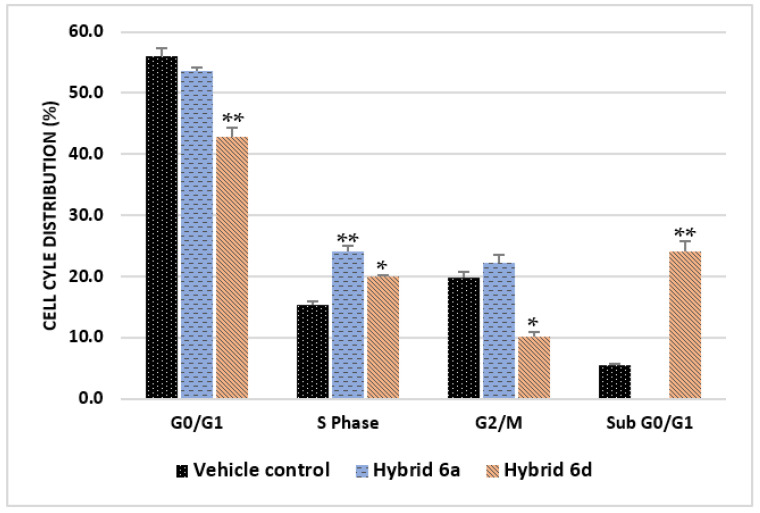
Cell cycle distribution after treatment with hybrids **6a** and **6d** in SW480 cells and representation of distribution percentages in bar chart form. All experiments were performed in duplicate and showed similar results. *p*-values lower than 0.05 were considered statistically significant (* *p* < 0.05; ** *p* < 0.01).

**Figure 7:**
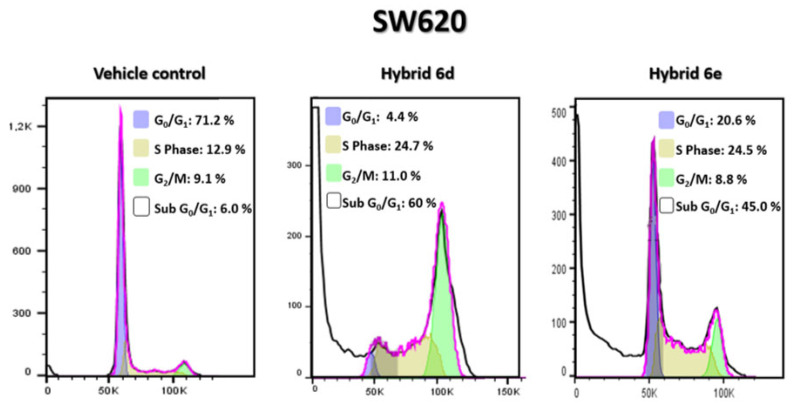

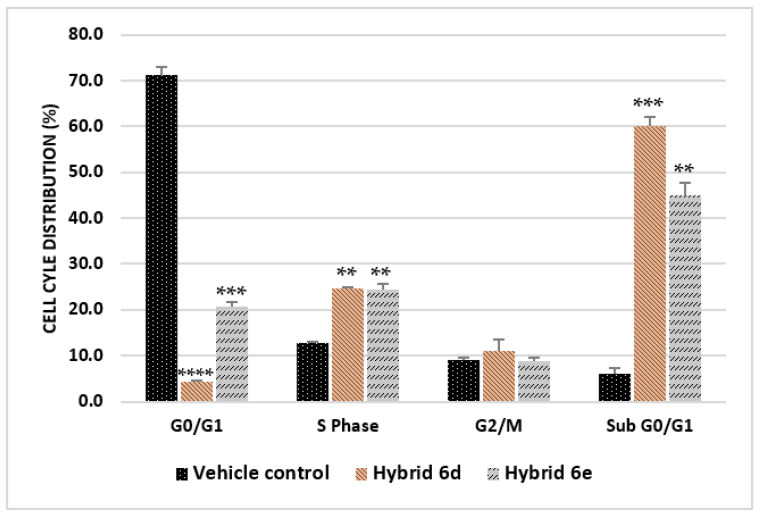
Cell cycle distribution after treatment with hybrids **6d** and **6e** in SW620 cells and representation of distribution percentages in bar chart form. All experiments were performed in duplicate and showed similar results. *p*-values lower than 0.05 were considered statistically significant (** *p* < 0.01; *** *p* < 0.001; **** *p* < 0.0001).

Cell lines SW480 and SW620 were treated with the previously obtained IC_50_ value, and their effect on cell cycle distribution was evaluated. The hybrid compound **6a** showed in cell line SW480 a significant increase in the cell population in the S phase of the cycle with a slight decrease in the population in the G0/G1 phase, but no significant changes in the G2/M and sub-G0/G1 phases. This could suggest an arrest in the cell cycle and the possible activity of this compound as a cytostatic agent. A different situation was presented with the hybrid **6d** in the SW480 cell line, which generated significant changes in all phases of the cycle at the expense of an increase in the sub-G0/G1 population with a corresponding decrease in the cell population in the G0/G1, S, and G2/M phases, suggesting that the cells suffered death after 48 h of treatment (Figure 6). The same situation was presented with this same hybrid compound and with compound **6e** when evaluated in the SW620 cell line, where both showed that more than 40% of the cell population was in the sub-G0/G1 phase (Figure 7). Similar findings were reported by Zhang et al., 2019 [34], with the hybrid compound **14** derived from the drugs 5-FU and Oxaliplatin. They reported its cytostatic activity by presenting cell cycle S-phase arrest against HCT-116 cells post-treatment. Similarly, Focaccetti et al., 2015 [38] confirmed the activity of 5-FU on the S phase of the cell cycle by inhibiting DNA synthesis due to a restriction in the availability of the enzyme thymidylate [38]. Other studies performed with the starting compound curcumin showed similar results to ours. Rahim et al. 2021 [39] reported that the DMCH analog of curcumin increased by more than 70% the sub-G0/G1 population in the cell cycle after treatment against HT29 colorectal adenocarcinoma cells and the metastatic SW620. The results of our study allowed us to demonstrate that the hybrid compounds **6a**, **6d**, and **6e** affect the cell cycle machinery, exerting their cytotoxic and cytostatic effects against SW480 and SW620 cells.

#### 3.2.4. Changes in Mitochondrial Membrane Potential (ΔΨm) Induced by 5-FU/Curcumin Hybrids

**Figure 8:**
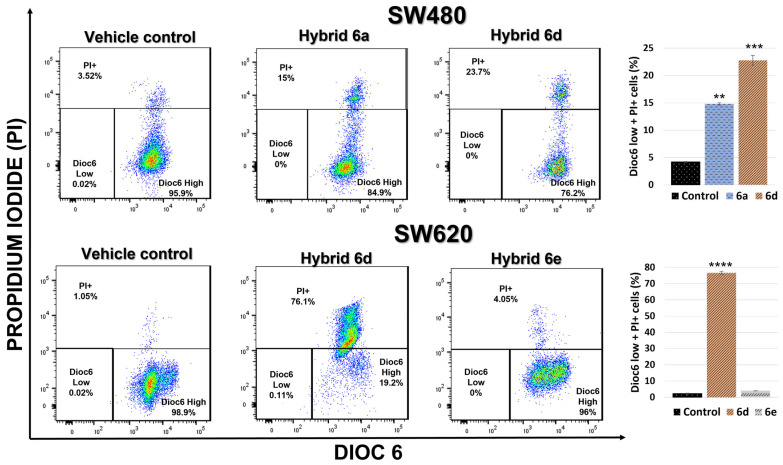
Changes in ΔΨm and plasma membrane integrity. Treatment with hybrids **6a** and **6d** in SW480 and hybrids **6d** and **6e** in SW620 cells. PI+: PI-positive cells, with membrane damage or dead cells; DiOC6 High: live cells with high membrane polarization; DiOC6 Low: cells in latency that lose membrane polarization. Data are presented as the mean of these two populations ± SE of three independent experiments. *p* values lower than 0.05 were considered statistically significant (** *p* < 0.01; *** *p* < 0.001; **** *p* < 0.0001).

Changes in the mitochondrial membrane potential are an indicator to assess the loss of mitochondrial integrity, which would reflect the onset of a proapoptotic signal [40]. According to the results obtained and illustrated in Figure 8, hybrid compounds **6a** and **6d** in the treatment against SW480 cells and hybrids **6d** and **6e** in the treatment against SW620 cells do not induce significant changes in the mitochondrial membrane potential; this was evidenced by not finding a cell population located in the lower left quadrant (low Dioc6). Additionally, in the treatments with the hybrid compounds **6a** and **6d** in SW480 cells, an increase in the PI+ population (upper quadrant) of 15% and 23.7%, respectively, was observed, as well as compound **6d** versus SW620 cells that concentrated most of their cells in this same quadrant (76.1%), which is indicative of dormant cells that lose the polarization of the plasma membrane, with damage to the membrane, or correspond to dead cells.

These results support the previous hypothesis in the analysis of the cell cycle distribution with hybrid **6a**, where a possible cytostatic effect was observed, while the other hybrids showed cytotoxic activity on the cell lines, possibly exerting a mitochondria-independent cell death mechanism. Other studies performed with curcumin-derived hybrid compounds reported a decrease in mitochondrial membrane potential in human cancer cells. Quan et al., 2018 [19] reported a significant decrease in mitochondrial membrane potential due to ROS accumulation in gastric cancer cells following time- and concentration-dependent treatment with the hybrid compound **7d**. In contrast, we reported in our research group with hybrids **4a** and **4g**, derived from 5-FU and genistein, that no significant changes in the ΔΨm were induced, supporting the hypothesis related to a possible cytostatic-like activity on SW480 and SW620 cells [17].

#### 3.2.5. Cell Death Induction by 5-FU/Curcumin Hybrids

**Figure 9:**
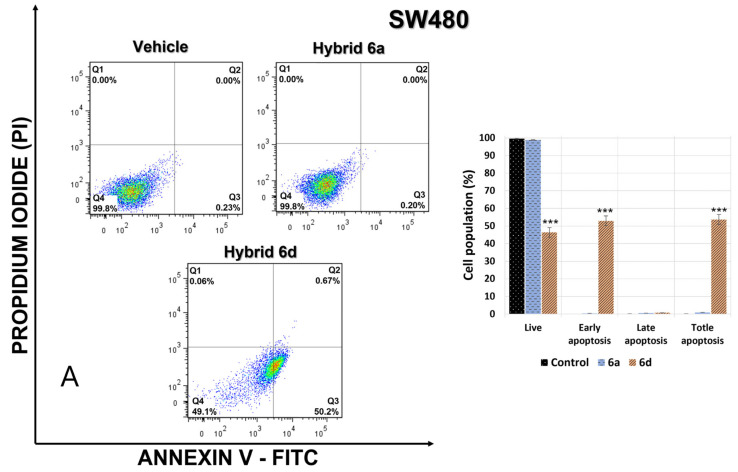

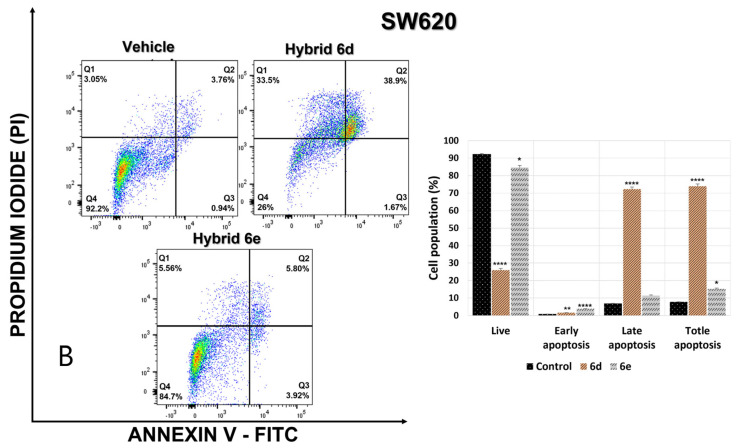
Apoptosis of SW480 (**A**) and SW620 (**B**) cells following treatment with the hybrid compounds **6a**, **6d**, and **6e**. The histograms represent one of at least two different experiments. Q1 + Q2: Cells that lost membrane integrity (late apoptotic cells, dead cells, necroptotic cells, secondary necrotic cells, others); Q3: Early apoptotic cells; Q4: Viable cells. Data are presented as the mean of these two populations ± SE of three independent experiments. *p*-values lower than 0.05 were considered statistically significant (* *p* < 0.05; ** *p* < 0.01; *** *p* < 0.001; **** *p* < 0.0001).

In the early stages of apoptosis, changes occur at the cell surface. One of these changes is the translocation of phosphatidylserine from the inner side of the plasma membrane to the outer layer, exposing it to the cell surface. Annexin V is a phospholipid-binding protein with a high affinity for phosphatidylserine, therefore, cells in early apoptosis show unique staining for Annexin V/FITC. After 48 h of exposure to the treatments with the hybrid compounds, it was observed that hybrid **6a** did not induce significant changes in the integrity of the plasma membrane of the SW480 cell line. These results were similar to those observed for the control, preserving the higher percentage of cells in the Q4 quadrant of viable cells. On the other hand, the hybrid compound **6d** against this same cell line induced a significant displacement of almost half of the cell population to the Q3 quadrant (50.2%), possibly implying a mechanism of cell death by apoptosis (early apoptotic cells), where the exposure of phosphatidylserine in the plasma membrane was evidenced without PI entering the cell (Figure 9A). This same compound **6d** against the SW620 cell line induced plasma membrane disintegration, as evidenced by the significative displacement of the cell population through the upper quadrant with positive staining for PI and Annexin V-FITC. Finally, treatment with compound **6e** concentrated most of its cell population (84.7%) in the Q4 quadrant of viable cells (negative for Annexin V-FITC and negative for PI) (Figure 9B). Other authors reported that the anticancer effect of curcumin on colorectal cancer cells is associated with the activation of the apoptosis pathway, involving multiple molecular targets [41]. Weng et al., 2015 [42] reported the treatment of SGC-7901 gastric cancer cells with curcumin-derived compounds, where the 5- and 28-hybrids significantly and dose-dependently increased cell apoptosis compared to negative control cells. Similarly, Ndreshkjana et al., 2019 [15] reported a significant increase in the fraction of apoptotic cells in 5-FU and thymoquinone-derived hybrid (SARB) treatments against HCT-116 cells.

#### 3.2.6. Determination of Apoptotic Biomarkers

**Figure 10:**
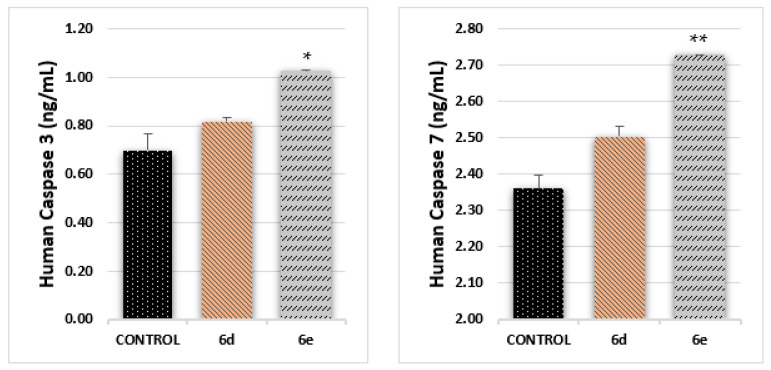
Effect of **6d** and **6e** hybrids on apoptotic biomarker expression in SW620 cells. Results are the mean ± SE of two independent experiments. *p*-values less than 0.05 were considered statistically significant (* *p* < 0.05; ** *p* < 0.01).

To complement the findings of the previous experiments, we proceeded to evaluate some biomarkers of importance in the process of cell death by apoptosis. The levels of caspase 3, caspase 7, caspase 8, and the tumor suppressor protein p53 were calculated after treatment with the hybrid compounds **6a**, **6d**, and **6e** in the SW480 and SW620 cell lines. Significantly increased levels of apoptotic cell death executioner caspases 3 and 7 were detected after treatment with hybrid compound **6e** in the SW620 cell line, which could be indicative of a possible mechanism of apoptotic cell death independent of the p53 status; however, further investigations are needed to confirm this hypothesis (Figure 10). Similarly, Watson et al., 2010 [43] and Ismail et al., 2019 [41] demonstrated that curcumin induces cell death by apoptosis in a p53 status-independent manner in colon cancer cells, highlighting its therapeutic potential in cancer treatment when there is resistance to chemotherapy due to defects in p53 expression or function. We also evaluated the same biomarkers in SW480 cells; however, we did not observe significant changes regarding the control. 

Regarding the other results, no significant differences were found in the levels of biomarkers after treatments in cells with hybrid compounds **6a** and **6d**; therefore, it may be inferred that hybrid compound **6a** presents a cytostatic rather than a cytotoxic effect and that compound **6d** induces a caspase-independent cell death process.

## 4. Conclusions

This study allowed us to demonstrate for the first time the chemopreventive potential of the hybrids synthesized from the natural compound curcumin and the reference anticancer drug 5-fluorouracil. The hybrid compounds exhibited cytotoxic activity against colon adenocarcinoma cells SW480 and its metastatic derivative SW620, with the most active compounds **6a**, **6d**, and **6e** having IC_50_ values ranging from 2.43–29.71 µM and 7.51–45.10 µM, respectively, for each cell line 48 h post-treatment. Furthermore, these compounds were selective when evaluated in the non-tumor cell lines HaCaT and CHO-K1, with better results than the starting compounds curcumin and 5-FU and their equimolar mixture. Under the conditions evaluated, we were able to demonstrate the antiproliferative effect of the hybrids and identify in compound **6a** its probable mechanism of cytostatic action against SW480 cells by analyzing the cell cycle effect and its S-phase arrest. Likewise, we were able to conclude that compound **6d** induced SW480 and SW620 cell death by a mitochondria- and caspases-independent death mechanism. Finally, compound **6e** induced significant upregulation of executioner caspases 3 and 7, suggesting a possible cell death by apoptosis; however, further experimental testing is required to clearly determine the death mechanisms associated with the evaluated hybrids.

All these findings highlight the chemopreventive potential of natural compounds in the molecular hybridization strategy, especially those inspired by curcumin in combination with different scaffolds such as the drug 5-FU, pointing out the importance of using them in further research, contributing to the design of new molecules with great potential and better selectivity compared to conventional chemotherapy.

## Figures and Tables

**Table 1:** Cytotoxic effect of 5-FU/Curcumin hybrids on SW480, HaCaT, and CHO-K1 cell lines.

Compound	IC_50_ (µM) CHO-K1		IC_50_ (µM) HaCaT		IC_50_ (µM) SW480		SI CHO-K1/SW480		SI HaCaT/SW480	
	24 h	48 h	24 h	48 h	24 h	48 h	24 h	48 h	24 h	48 h
**6a**	82.84 ± 6.43	73.70 ± 1.07	71.77 ± 4.22	27.90 ± 2.53	75.30 ± 4.65	**17.37 ± 1.16**	1.10	**4.24**	0.95	**1.61**
**6b**	51.34 ± 2.86	46.50 ± 1.34	58.07 ± 3.30	18.45 ± 1.10	48.36 ± 2.82	17.14 ± 0.80	1.06	2.71	1.20	1.07
**6c**	26.30 ± 2.99	30.54 ± 2.18	45.44 ± 4.18	19.10 ± 1.68	30.42 ± 2.69	16.39 ± 0.97	0.86	1.86	1.49	1.17
**6d**	12.43 ± 1.34	28.10 ± 1.95	42.64 ± 6.93	20.32 ± 2.66	15.73 ± 1.91	**2.43 ± 0.33**	0.79	**11.56**	2.71	**8.36**
**6e**	33.80 ± 2.20	34.51 ± 1.37	76.86 ± 5.87	19.65 ± 1.99	38.79 ± 5.46	18.18 ± 0.88	0.87	1.90	1.98	1.08
**6f**	>100	>100	>100	35.10 ± 4.74	>100	29.71 ± 2.43	NA	>3	NA	1.18
**6g**	>100	>100	>100	>100	>100	>100	NA	NA	NA	NA
**6h**	>100	>100	>100	>100	>100	>100	NA	NA	NA	NA
Mixture 5FU + Curcumin *	87.64 ± 12.55	17.66 ± 2.28	88.63 ± 6.37	7.83 ± 1.30	95.92 ± 3.37	27.23 ± 1.21	0.91	0.65	0.92	0.44
**Curcumin**	20.18 ± 2.41	36.29 ± 4.17	>100	28.78 ± 2.33	84.64 ± 6.60	90.44 ± 1.00	0.24	0.40	>1	0.32
**5-FU**	543.50 ± 52.94	173.20 ± 14.61	>1000	118.67 ± 2.84	1544.01 ± 127.90	174.30 ± 19.10	0.35	0.99	NA	0.68

IC_50_ values were obtained from dose-response curves for each compound evaluated. The selectivity index (SI) was calculated using the IC_50_ values in non-malignant cells (CHO-K1 and HaCaT) relative to the IC_50_ of SW480 cells. The values in bold indicate the best results obtained. Data are represented as the mean ± standard deviation of at least three independent experiments. NA: not applicable. * Equimolar combination.

**Table 2:** Cytotoxic effect of 5-FU/Curcumin hybrids on SW620, HaCaT, and CHO-K1 cell lines.

Compound	IC_50_ (µM) CHO-K1		IC_50_ (µM) HaCaT		IC_50_ (µM) SW620		SI CHO-K1/SW620		SI HaCaT/SW620	
	24 h	48 h	24 h	48 h	24 h	48 h	24 h	48 h	24 h	48 h
**6a**	82.84 ± 6.43	73.70 ± 1.07	71.77 ± 4.22	27.90 ± 2.53	>100	45.10 ± 0.98	<1	1.63	<1	0.62
**6b**	51.34 ± 2.86	46.50 ± 1.34	58.07 ± 3.30	18.45 ± 1.10	90.21 ± 9.33	33.73 ± 1.03	0.57	1.38	0.64	0.55
**6c**	26.30 ± 2.99	30.54 ± 2.18	45.44 ± 4.18	19.10 ± 1.68	83.90 ± 8.08	28.07 ± 1.85	0.31	1.09	0.54	0.68
**6d**	12.43 ± 1.34	28.10 ± 1.95	42.64 ± 6.93	20.32 ± 2.66	37.08 ± 3.05	**7.51 ± 1.47**	0.34	**3.74**	1.15	**2.71**
**6e**	33.80 ± 2.20	34.51 ± 1.37	76.86 ± 5.87	19.65 ± 1.99	>100	**14.52 ± 1.31**	<1	**1.77**	<1	**1.36**
**6f**	>100	>100	>100	35.10 ± 4.74	NI	>100	NA	NA	NA	<1
**6g**	>100	>100	>100	>100	NI	>100	NA	NA	NA	NA
**6h**	>100	>100	>100	>100	NI	>100	NA	NA	NA	NA
Mixture 5FU + Curcumin *	87.64 ± 12.55	17.66 ± 2.28	88.63 ± 6.37	7.83 ± 1.30	>100	63.92 ± 2.05	<1	0.28	<1	0.12
**Curcumin**	20.18 ± 2.41	36.29 ± 4.17	>100	28.78 ± 2.33	163.60 ± 5.88	22.21 ± 2.37	0.12	1.63	NA	1.30
**5-FU**	543.5 ± 52.94	173.20 ± 14.61	>1000	118.67 ± 2.84	898.80 ± 60.74	180.90 ± 18.80	0.60	0.96	<1	0.66

IC_50_ values were obtained from dose-response curves for each compound evaluated. The selectivity index (SI) was calculated using the IC_50_ values in non-malignant cells (CHO-K1 and HaCaT) relative to the IC_50_ of SW620 cells. The values in bold indicate the best results obtained. Data are represented as the mean ± standard deviation of at least three independent experiments. NI: not inhibition. NA: not applicable. * Equimolar combination.

**Table 3:** Data of viability (%) at the lowest and the highest concentrations evaluated.

Cell Line	Hybrids	Concentration (µM)	Time after Plating (Days)			
			2	4	6	8
			Cell Viability (%) ± SE			
SW480	**6a**	2.5	97.91 ± 1.55	81.21 ± 2.80	59.53 ± 3.45	42.83 ± 1.30
		40	16.24 ± 2.51	8.00 ± 1.81	2.04 ± 0.35	0.00
	**6d**	0.625	90.11 ± 1.45	55.47 ± 2.21	46.36 ± 0.18	32.34 ± 0.77
		10	7.35 ± 1.45	0.75 ± 0.20	0.68 ± 0.14	0.00
SW620	**6d**	1.25	90.95 ± 2.85	85.19 ± 3.23	67.34 ± 2.32	54.41 ± 2.76
		20	42.47 ± 4.57	7.10 ± 0.95	0.00	0.00
	**6e**	2.5	95.57 ± 4.08	60.65 ± 0.46	56.53 ± 2.09	48.93 ± 3.46
		40	8.77 ± 1.85	0.00	0.00	0.00

## Data Availability

Data are contained within the article.
